# Publisher’s Note: *Children* and Sustainability

**DOI:** 10.3390/children1010003

**Published:** 2013-10-18

**Authors:** Shu-Kun Lin

**Affiliations:** MDPI AG, Postfach, CH-4005 Basel, Switzerland; E-mail: lin@mdpi.com

We are launching the open access journal *Children*, a scholarly forum with pediatrics as the main focus. The journal title came to the minds of Dr. Brietta Pike and I in 2011, when both of us were lucky enough to have a new baby in our respective families. 

As the publisher of MDPI journals, over the past few years I have launched many journals related to sustainability [1] — now yet one more journal, *Children*. 

When the older generation passes away, we will be survived by our offspring. The people of the present generation may become enemies of each other due to great differences. However, Romeo and Juliet—the children of two hostile families—became lovers after only one generation. Notably, the black African and Caucasian races in America have started to intermarry and have already produced many children—one of them, a boy called Barack Obama, is the current President of the United States of America. If the human race is still thriving in 10,000 years [2], each of us alive in the present could be the antecedents of all children after interracial, intercultural, and international marriages over many generations. This might be a good reason for all of us living now to tolerate the differences (or better termed, diversity) and to work for the common wellbeing of the children of many future generations: to preserve nonrenewable resources, to protect the ecosystem, to continue our diverse traditions, and to carry on the virtues of our ancestors. 

This means that everyone living now can be considered as family relatives, considering the mixing process likely to take place in future generations. We have a great deal to do together to ensure that all the children of the present generation fulfill their potential. We need to take great care of the health of all children of our present generation. This journal will focus on these issues and others related to childhood. I hope that you enjoy publishing with us. 
